# Description of Cumbeba (*Tacinga inamoena*) Waste Drying at Different Temperatures Using Diffusion Models

**DOI:** 10.3390/foods9121818

**Published:** 2020-12-07

**Authors:** João P. L. Ferreira, Wilton P. Silva, Alexandre J. M. Queiroz, Rossana M. F. Figueirêdo, Josivanda P. Gomes, Bruno A. Melo, Dyego C. Santos, Thalis L. B. Lima, Rodolfo R. C. Branco, Ihsan Hamawand, Antonio G. B. Lima

**Affiliations:** 1 Federal University of Campina Grande, Campina Grande 58428-830, Brazil; Wiltonps@uol.com.br (W.P.S.); alexandrejmq@gmail.com (A.J.M.Q.); rossanamff@gmail.com (R.M.F.F.); josivanda@gmail.com (J.P.G.); b.amelo@yahoo.com (B.A.M.); tthallisma@gmail.com (T.L.B.L.); rodolfo.castelobranco@outlook.com (R.R.C.B.); antonio.gilson@ufcg.edu.br (A.G.B.L.); 2 Federal Institute of Acre, Xapuri 69930-000, Brazil; dyego.csantos@gmail.com; 3 University of Southern Queensland, Toowoomba, QLD 4350, Australia; ihsan.hamawand@gmail.com

**Keywords:** by-product, drying kinetics, analytical solution, effective mass diffusivity, convective mass transfer coefficient, activation energy

## Abstract

One approach to improve sustainable agro-industrial fruit production is to add value to the waste generated in pulp extraction. The processing of cumbeba (*Tacinga inamoena*) fruits generates a significant amount of waste, which is discarded without further application but can be a source of bioactive compounds, among other nutrients. Among the simplest and most inexpensive forms of processing, convective drying appears as the first option for the commercial utilization of fruit derivatives, but it is essential to understand the properties of mass transfer for the appropriate choice of drying conditions. In this study, cumbeba waste was dried at four temperatures (50, 60, 70 and 80 °C). Three diffusion models were fitted to the experimental data of the different drying conditions. Two boundary conditions on the sample surface were considered: equilibrium condition and convective condition. The simulations were performed simultaneously with the estimation of effective mass diffusivity coefficients (*D_ef_*) and convective mass transfer coefficients (*h*). The validation of the models was verified by the agreement between the theoretical prediction (simulation) and the experimental results. The results showed that, for the best model, the effective mass diffusivities were 2.9285 × 10^−9^, 4.1695 × 10^−9^, 8.1395 × 10^−9^ and 1.2754 × 10^−8^ m^2^/s, while the convective mass transfer coefficients were 6.4362 × 10^−7^, 8.7273 × 10^−7^, 8.9445 × 10^−7^ and 1.0912 × 10^−6^ m/s. The coefficients of determination were greater than 0.995 and the chi-squares were lower than 2.2826 × 10^−2^ for all simulations of the experiments.

## 1. Introduction

Cumbeba (*Tacinga inamoena*) is a cactus species typical of northeastern Brazil, which produces fruits that have been reported as a source of bioactive compounds, such as phenolic compounds, carotenoids, betalains and vitamin C [1,2,3,4,5,6,7]. Cumbeba processing generates a large volume of waste, representing from 64.70% to 79.01% (*w*/*w*) of the processed fruit [1,2,6], which is discarded without further applications. However, the processing of fruit and vegetable wastes for the production of higher-added-value products, such as antioxidant compounds, natural dyes, dietary fibers, starch, edible oils and enzymes [8,9,10,11,12,13], has the potential to become an important agribusiness segment, which can be used as an alternative to increase profits in fruit production and, at the same time, represent an ecologically correct strategy to minimize environmental pollution.

Fresh cumbeba waste is highly perishable, so drying is a critical step in processing to increase its shelf life for subsequent use. Therefore, it is important to study the characteristics of the drying process to obtain the desired amount of waste with the lowest energy demand and best quality possible. In this context, the modeling of the drying process stands out. Studies on mathematical modeling of the waste drying process generally use an empirical approach, based on models such as those of Page, Thompson and Henderson, among others [14,15,16,17,18]. However, the description of drying with empirical models sacrifices the investigation of important issues, particularly those related to the influence of drying conditions on the properties of mass transport (water), the understanding of the formation of resistances inside and at the boundary of the material, and the resulting consequences on water removal dynamics.

These limitations can be overcome with simulations using theoretical models, which are based on physical principles and, therefore, enable a more realistic description of the drying process, including graphical and quantitative information on how internal water distribution evolves [19,20,21]. Most of the theoretical models used to study drying problems are mainly based on Fick’s second law, mathematically represented by the diffusion equation [22,23,24]. Generally, for simple geometries and under the assumption of simplifying hypotheses, the diffusion equation is solved by analytical methods [22,23]. Despite the restrictions, simulation with analytical models is a useful tool for monitoring, controlling and optimizing the drying process and, additionally, it is also used to generate validation data for numerical simulations [25].

In several studies available in the literature on waste drying, mass diffusivity is determined by nonlinear regression, using only the first term of the infinite series of the analytical solution of the diffusion equation, solved for equilibrium boundary conditions [26,27,28,29,30,31]. However, this is particularly problematic, because the underlying modeling of the drying process using this approach may result—for high mass transfer Biot numbers characteristic of the equilibrium boundary conditions—in significant truncation errors, especially in the initial instants of drying [32,33,34]. Thus, mathematical models that incorporate an appropriate number of terms from the analytical solution of the diffusion equation and/or the convective boundary condition to describe the drying process are more successful [33,35,36,37,38].

In this context, the present study aimed to simulate the drying process of cumbeba waste, arranged in the form of a flat slab and dried at operating temperatures of 50, 60, 70 and 80 °C, using diffusion models with equilibrium and convective boundary conditions, as well as to determine the properties of mass transport, such as mass diffusivity and convective mass transfer coefficients and activation energy during the drying process.

## 2. Materials and Methods

### 2.1. Material

Ripe cumbeba (*Tacinga inamoena*) fruits, with yellow-orange peel, were harvested in the municipality of Afogados da Ingazeira (Pernambuco, Brazil—7°45′3″ S latitude, 37°38′20″ W longitude, 525 m altitude). Fruits with no defects and/or injuries were washed with neutral detergent and a soft bristle brush to eliminate the glochids (spines) and sanitized by immersion in chlorinated solution (100 mg/kg) for 20 min; subsequently, they were rinsed with drinking water to remove excess sanitizing solution and then passed through the pulp extractor (Laboremus, Brazil), where the by-product (cumbeba waste) was collected and used as raw material in this study. The cumbeba waste, composed of fractions of peel and traces of pulp and seeds, was mixed to ensure a better homogenization and uniform distribution of the fractions, and then packed in low-density polyethylene bags with capacity of 1.0 kg and immediately stored at −18 °C in a freezer (Hesstar, HVF-301S, Brazil), where it remained until use in the experimental steps. The moisture content of the waste was determined gravimetrically by drying in an oven at 70 °C and pressure ≤ 100 mmHg until reaching constant mass, according to the standard method 934.01 of the Association of Official Analytical Chemists (AOAC) [39].

### 2.2. Convective Drying of the Waste

Prior to the drying experiments, the required amount of cumbeba waste was thawed under refrigeration (≈4 °C for 24 h), left at room temperature (≈25 °C) for 2 h and subjected to crushing in a food processor (Philco, PMP1600P model, Brazil) for 5 min in the ‘high’ mode (rotation speed level: 2). The crushed waste was distributed on circular aluminum trays (81.1 ± 0.1 mm in diameter) while a light pressure was applied to form slabs of uniform thickness (9.555 ± 0.436 mm) and dried in an oven with forced air circulation (Fanem, 320 model, Brazil) at four temperatures (50, 60, 70 and 80 °C) and air speed of 1.5 m/s. The masses of the samples during the experiments were measured at time intervals ranging from 5 min at the beginning of drying to 60 min at the end of the process, on a digital scale with accuracy of 0.01 g (Marte, AS5500C model, Brazil). The drying experiments were conducted until the samples reached constant mass, that is, when there was no mass variation in three consecutive weighing procedures, which was assumed to be the state of equilibrium. After drying, the dry matter was obtained, and the water content was calculated at each drying time according to the AOAC [39]. All drying experiments were carried out with three replicates.

### 2.3. Mathematical Modeling of Drying Kinetics

#### 2.3.1. Diffusion Equation or Governing Equation

To describe a drying process, the one-dimensional diffusion equation for an infinite slab can be written in the form of Equation (1) [22,23]:

(1) $$\frac{\partial M}{\partial t}\;=\;\frac{\partial }{\partial x}\;\left(D_{ef}\frac{\partial M}{\partial x}\right)$$

where *M* is the moisture content on dry basis (d.b.), *Def* is the effective mass diffusivity (m^2^/s), t is the time (s) and *x* is the Cartesian coordinate of the position (m). Equation (1) was solved to describe the drying of cumbeba waste considering the following premises: (1) absence of volume contraction during drying; (2) uniform initial distribution of water; (3) diffusion is the only mechanism of water transport inside the sample; (4) the waste is considered homogeneous and isotropic; (5) effective mass diffusivity does not vary during the process; (6) convective mass transfer coefficient is constant during diffusion; (7) the process is considered isothermal. Different assumptions in the description of the drying process result in different solutions of Equation (1); therefore, in the present study, three models were used to describe the drying of cumbeba waste, and their solutions are presented below.

##### Model 1: Analytical Solution for the Diffusion Equation Considering Third-Kind or Convective Boundary Condition

The boundary condition of the third kind is expressed considering that the internal diffusive mass flow at the boundary of the product is equal to the external convective flow in the vicinity of this boundary. Thus, for an infinite slab, this consideration results in Equation (2):

(2) $${-\;D}_{ef}{\frac{\partial M\;\left(x,t\right)}{\partial x}|}_{x\;=\;\pm \;L/2}\;=\;h\left[{M\left(x,\;t\right)|}_{x\;=\;\pm \;L/2}-{\;M}_{eq}\;\right],\;t\;>\;0$$

In Equation (2), *h* is the convective mass transfer coefficient (m/s), *M*(*x,t*) is the value of moisture content at position *x* at instant *t*, *M_eq_* is the equilibrium moisture content and *L* is the infinite slab thickness (m). For the premises mentioned above (Section 2.3.1), with the initial moisture content indicated by *M*_0_ and the boundary condition defined by Equation (2), the analytical solution *M*(*x*,*t*) of Equation (1) is given by Equation (3) [22,23]:

(3) $$M\left(x,\;t\right){\;=\;M}_{eq}{\;+\;(M}_{0}{\;-\;M}_{eq})\overset{\infty }{\underset{n\;=\;1}{\sum }}A_{n}\times \;cos\left(\mu_{n}\frac{x}{L/2}\right)exp\left(-\frac{\mu_{n}{}^{2}}{{\left(L/2\right)}^{2}}D_{ef}t\right)$$

where the *x*-axis origin is located at the central point of the infinite slab. In Equation (3), coefficient *A_n_* is given by Equation (4):

(4) $$A_{n}\;=\;\frac{{4sin\;\mu }_{n}}{{2\mu }_{n}{\;+\;sin(2\mu }_{n})}$$

where *μ_n_* are the roots of the characteristic equation for an infinite slab:

(5) $${cot\;\mu }_{n}\;=\;\frac{{\;\mu }_{n}}{Bi}$$

In Equation (5), *Bi* is the mass transfer Biot number, given by Equation (6):

(6) $$Bi\;=\;\frac{hL/2}{D_{ef}}$$

where *L* is the characteristic length (m). The expression to determine the average moisture content, $\bar{M}\left(t\right)$at an instant *t*, is in the form of Equation (7):

(7) $$\bar{M}\left(t\right){\;=\;M}_{eq}\;+\;\left(M_{0}\;-{\;M}_{eq}\right)\overset{\infty }{\underset{n\;=\;1}{\sum }}B_{n}exp\left(-\frac{\mu_{n}{}^{2}}{{\left(L/2\right)}^{2}}D_{ef}t\right)$$

where the parameter *B_n_* is given by Equation (8):

(8) $$B_{n}\;=\;\frac{{2Bi}^{2}}{\mu_{n}{}^{2}{\;(Bi}^{2}{\;+\;Bi\;+\;\mu }_{n}{}^{2})}$$

Equation (5) is a transcendental equation that can be solved for a specified mass transfer Biot number. The first 16 roots of Equation (5) were calculated for 469 specified values of mass transfer Biot number, from *Bi* = 0 (which corresponds to an infinite resistance to water flow on the surface) to *Bi* = 200 (which practically corresponds to the equilibrium boundary condition). In the literature it is common to find Equation (7) rewritten to express the value of the dimensionless moisture content, given by Equation (9):

(9) $$MR\left(t\right)\;=\;\frac{\bar{M}\left(t\right)\;-{\;M}_{eq}}{M_{0}\;-{\;M}_{eq}}\;=\;\overset{\infty }{\underset{n\;=\;1}{\sum }}B_{n}exp\left(-\frac{\mu_{n}{}^{2}}{{\left(L/2\right)}^{2}}D_{ef}t\right)$$

##### Model 2: First-Kind or Prescribed Boundary Condition

For the first-kind boundary condition, an equilibrium condition was assumed on the sample surface, so that the moisture content on the surface is equal to the equilibrium moisture content. Thus, for an infinite slab, this imposition results in Equation (10):

(10) $${M|}_{x\;=\;\pm \;L/2\;}{\;=\;M}_{eq},\;t\;>\;0$$

For an infinite homogeneous slab of thickness *L*, with initially uniform distribution of moisture content (*t* = 0, *M* = *M*_0_, 0 ≤ *x* ≤ *L*) and equilibrium moisture content *M_eq_*, the solutions of Equation (1) are also given by Equations (3) and (7). However, in this case where the mass transfer Biot number tends to infinity, characteristic of the boundary condition of the first kind (*Bi* >> 0), Equation (5) is given by Equation (11) [40]:

(11) $${\cot\mu }_{n}\;=\;0$$

and, as a consequence, *μ_n_* is given by Equation (12):

(12) $$\mu_{n}\;=\;\frac{\pi (2n\;-\;1)}{2}$$

With *n* = 1, 2, 3..., ∞. Thus, the coefficient *A_n_* (Equation (4)) is defined and the local moisture content at any time, *M*(*x*,*t*), can be calculated using Equation (3). On the other hand, when the mass transfer Biot number tends to infinity B_n_, calculated with Equation (8), it is redefined according to Equation (13):

(13) $$B_{n}\;=\;\frac{2}{\mu_{n}{}^{2}}$$

With the coefficient *B_n_* calculated using Equation (13), the average moisture content at the instant t, $\bar{M}\left(t\right)$, is given in the form of Equation (14) [22]:

(14) $$\bar{M}\left(t\right){\;=\;M}_{eq}\;+\;\left(M_{0\;}{-\;M}_{eq}\right)\frac{8}{\pi^{2}}\overset{\infty }{\underset{n\;=\;0}{\sum }}\frac{1}{{\left(2n\;+\;1\right)}^{2}}exp\left[-{\left(2n\;+\;1\right)}^{2}\pi^{2}D_{ef}\frac{t}{L^{2}}\right]$$

where $\bar{M}\left(t\right)$ is the average moisture content (d.b.) at time *t*, *M_eq_* is the moisture content for *t* >> 0; *M*_0_ is the moisture content at *t* = 0, *D_ef_* is the effective mass diffusivity (m^2^/s), *t* is the time (s) and *L* is the thickness of the infinite slab (m). From Equation (14), the dimensionless moisture content is calculated in the form of Equation (15):

(15) $$MR\left(t\right)\;=\;\frac{\bar{M}\left(t\right)\;-{\;M}_{eq}}{M_{0\;}-{\;M}_{eq}}\;=\;\frac{8}{\pi^{2}}\overset{\infty }{\underset{n\;=\;0}{\sum }}\frac{1}{{\left(2n\;+\;1\right)}^{2}}exp\left[-{\left(2n\;+\;1\right)}^{2}\pi^{2}D_{ef}\frac{t}{L^{2}}\right]$$

##### Model 3: First Term of the Infinite Series

Model 3 is one-dimensional, with no external resistance, and assumes that the geometric shape of the sample is an infinite slab (area >> thickness). The one-dimensional analytical solution of Equation (1) for these conditions, in terms of the dimensionless moisture content, i.e., Equation (15), is simplified to only the first term of the series, which follows the approach used in several studies on waste drying [26,27,30,31,41,42]. In this case, Equation (15) is rewritten as follows:

(16) $$MR\left(t\right)\;=\;\frac{\bar{M}\left(t\right)\;-{\;M}_{eq}}{M_{0\;}{-\;M}_{eq}}\;=\;\frac{8}{\pi^{2}}exp\left(-\pi^{2}D_{ef}\frac{t}{L^{2}}\right)$$

### 2.4. Computer Simulation

As mentioned previously (Section 2.3.1), different models were tested, and to determine the best model for describing the drying of cumbeba waste the coefficient of determination (R^2^) and chi-square (χ^2^) of the simulations were used as quality-of-fit criteria. According to [43,44], the model with the highest R^2^ value and the lowest χ^2^ value is the best to describe the drying kinetics of the product.

The simulations were performed, in the case of the convective boundary condition (model 1), using Convective Adsorption–Desorption software version 3.2 (Federal University of Campina Grande, PB, Brazil), and for the equilibrium boundary condition (model 2), using Prescribed Adsorption–Desorption software version 2.2 (Federal University of Campina Grande, PB, Brazil), which are programs that determine the transport parameters, i.e., the optimal values of the effective mass diffusivity (*D_ef_*) and/or the convective mass transfer coefficient (*h*) using an optimizer coupled to the analytical solution of the diffusion equation, through known experimental data. Briefly, the optimizer scans the entire domain of the values of mass transfer Biot number (*Bi*) and/or effective mass diffusivity (*D_ef_*) and finds the minimum for the objective function $\chi^{2\;}\;=\;\overset{N_{p}}{\underset{i\;=\;1}{\sum }}{\left[{\bar{MR}}_{i}{}^{exp}\;-\;{\bar{MR}}_{i}{}^{ana}{(D}_{ef},\;Bi)\right]}^{2}\frac{1}{\sigma_{i}{}^{2}}$, which is the chi-square (χ^2^), defined according to [45,46]. As the uncertainties associated with the experimental points (*σ_i_*) were not initially determined and were therefore unknown, *σ_i_* = 1 was attributed to all experimental points and thus the same statistical weight (1/*σ_i_* = 1) was attributed to all of them. The source codes were compiled by Compaq Visual Fortran (CVF) 6.6.0 Professional Edition, using the QuickWin Application programming option on the Windows Vista platform. The convergence criterion stipulated for the determination of effective mass diffusivity for each specified mass transfer Biot number was 1 × 10^−15^. The software programs were developed by the second author of this study and is available for evaluation at www.labfit.net/Convective.htm and at www.labfit.net/Prescribed.htm (Convective Adsorption–Desorption and Prescribed Adsorption–Desorption, respectively). The graphs showing the drying kinetics, parameterized in terms of the dimensionless moisture content, were also obtained through the software programs used to determine *D_ef_* and/or *h* and simulate the process. For more information on the development of the software programs used in this study, such as the algorithm for optimizing transport parameters, *h* and/or *D_ef_*, readers should consult the works of [32,33] and their references.

The software programs employed in the simulation of models 1 and 2 (Convective Adsorption–Desorption and Prescribed Adsorption–Desorption, respectively) were used for the solution of the diffusion equation (Equation (1)), in addition to the assumptions presented in Section 2.3.1, a condition of axial symmetry (*t* > 0; 0 ≤ *r* ≤ *R*; ${\partial M/\partial x|}_{x\;=\;\pm \;L/2\;}\;=\;0$. As a consequence, the origin of the reference system (*x*-axis) is considered to be located at the central point of the infinite slab (*x* = *L*/2). However, this is not consistent with the physical reality of the drying of cumbeba waste, because the origin of its reference system (*x*-axis) is located at the aluminum tray-waste interface (*x* = 0). Thus, to overcome this problem without the need for implementing new source codes, it was only necessary to double the value of the sample thickness, because this artifice caused the central point of the infinite slab, during the execution of the solution of the diffusion equation by the software programs, to coincide with the origin of the *x*-axis of the real problem.

For model 3, a simple nonlinear regression made it possible to determine the effective mass diffusivity (*D_ef_*). The simulation was performed in Statistica^®^ software version 7.0 (StatSoft^®^ Inc. United States).

### 2.5. Arrhenius Equation

To relate the effective mass diffusivity (*D_ef_*) and drying temperature (*T*), an Arrhenius-type equation, Equation (17), can be used [34,47,48,49]:

(17) $$D_{ef}{\;=\;D}_{0}\;exp\left[-\frac{E_{a}}{R\left(T\;+\;273.15\right)}\right]$$

In Equation (17), *D*_0_ is the pre-exponential factor (m^2^/s), *E_a_* is the activation energy (kJ/mol), *R* is the universal constant of gases (0.008314 kJ/mol K) and *T* is the drying air temperature (°C). On the other hand, an Arrhenius-type equation (Equation (18)) can also be used to relate the convective mass transfer coefficient (*h*) and the drying temperature (*T*) [50,51,52,53]:

(18) $$h\;=\;A\;exp\left[-\frac{B}{R\left(T\;+\;273.15\right)}\right]$$

where *A* and *B* are fitting parameters.

## 3. Results and Discussion

Initially, the result obtained with model 3 will be presented. Then, the result obtained with model 2 will be presented and finally the result obtained with model 1. At the beginning of the process (*t* = 0 and *MR* = 1), the amount of water in cumbeba waste was 403.242 ± 26.310% d.b. (80.079 ± 1.039% w.b.). The moisture content values observed in the equilibrium (*t* = *t*_∞_ and *MR* = 0) in the tests carried out at temperatures of 50, 60, 70 and 80 °C were 6.601, 5.297, 10.431 and 10.095% d.b., with drying times of 1560, 1320, 1080 and 780 min, respectively.

### 3.1. Results Obtained with Model 3

For model 3, the results obtained in the simulation for all temperatures are presented in Table 1. Statistical indicators (R^2^ ≤ 0.9442 and χ^2^ ≥ 2.2856), which can be considered poor, indicate that model 3 did not fit well to the experimental data of cumbeba waste drying.

**Figure 1:**
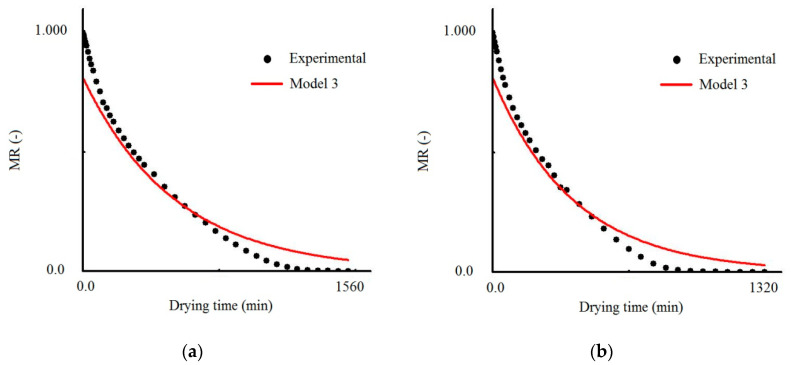

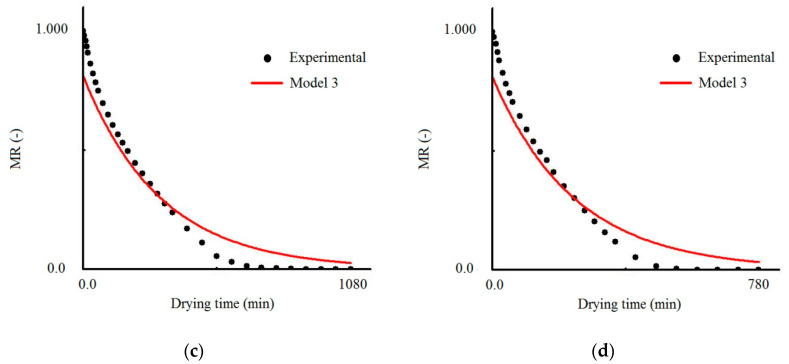
Comparison of dimensionless moisture contents obtained in the experiment and predicted using model 3 at drying air temperatures of (**a**) 50 °C, (**b**) 60 °C, (**c**) 70 °C and (**d**) 80 °C.

Figure 1 shows the simulated curves fitted to the experimental data set for drying air temperatures of 50, 60, 70 and 80 °C. As an example, for the drying air temperature of 50 °C (Figure 1a), at *t* = 0, the dimensionless moisture content is about 0.81, instead of 1.0, as expected, confirming the low quality of the fit of model 3 to the experimental data. This result can be generalized to the other temperatures investigated (see Figure 1b–d), enabling the conclusion that model 3 should be avoided for describing the drying of cumbeba waste. According to [34], for low mass transfer Biot numbers, some terms of the infinite series representing the solution of the diffusion equation are sufficient to obtain results with truncation errors that can be considered insignificant. However, according to the authors, when the Biot number increases, it is necessary to significantly increase the number of terms of the infinite series to avoid major truncation errors, especially at the initial instants of the drying process. In this case, in the fit of the analytical solution with only the first term of the infinite series, as in the case of model 3, first experimental points should be removed to minimize errors in the determination of transport properties [54,55] and, therefore, in the description of the drying process of the product. However, in this work, no point was removed to describe the drying kinetics of this process, showing how poor this model is, as is seen in Figure 1. As an example, it can be observed that, for *t* = 0, the moisture content obtained through the model 3 is about 0.81, instead of the expected value, which is 1.0.

### 3.2. Results Obtained with Model 2

For the boundary condition of the first kind (model 2), whose simulation results are presented in Table 2, the Biot number, given by *Bi* = (*hL*/2)/*D_ef_*, tends to infinity, and the only parameter to be determined is the effective mass diffusivity (*D_ef_*). The *D_ef_* values obtained for convective drying of cumbeba waste ranged from 1.1285 × 10^−9^ m^2^/s (50 °C) to 2.5368 × 10^−9^ m^2^/s (80 °C) (Table 2). This behavior (increase in *D_ef_* with the increment in temperature) is explained by the increase in the rate of heat transfer between the waste and the drying air, which occurs due to the increase in temperature [56], which results in greater kinetic energy of water molecules [57,58], an increase of vapor pressure in the sample [59] and greater diffusion towards the external layers of the waste. Comparable results of change in *D_ef_* with temperature have been observed in the literature by several authors for different types of waste, such as grape seeds [60], olive-waste cake [61], okara (by-product obtained during the production of soy milk and tofu) [62], passion fruit peel [63] and olive pomace waste [64].

**Figure 2:**
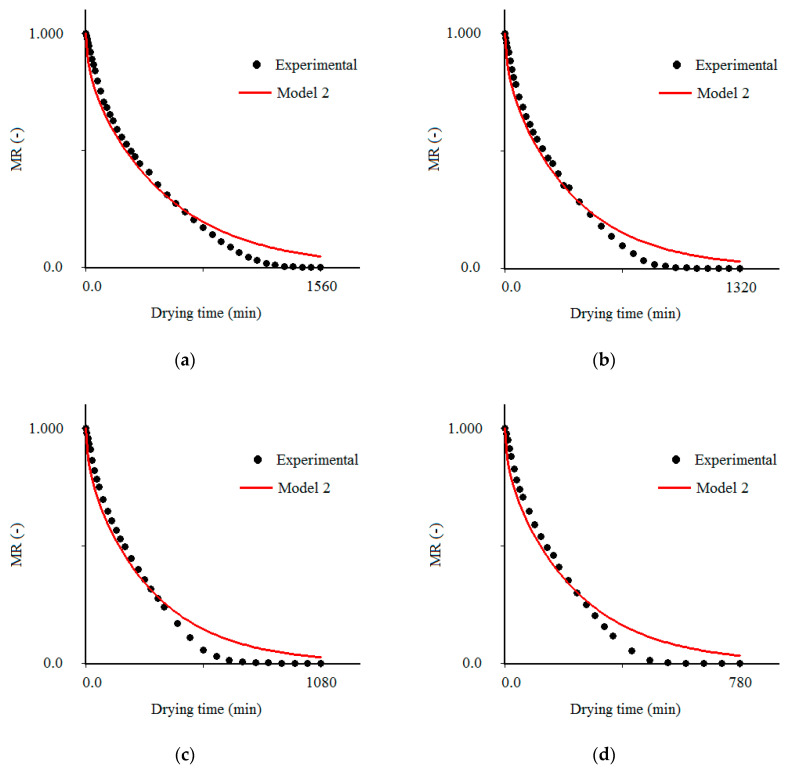
Comparison of dimensionless moisture contents obtained in the experiment and predicted using model 2 at drying air temperatures of (**a**) 50 °C, (**b**) 60 °C, (**c**) 70 °C and (**d**) 80 °C.

After determining the value of *D_ef_*, the cumbeba waste drying kinetics could then be described by model 2, as shown in Figure 2. The results obtained with model 2 (Table 2) can be considered better than those obtained with model 3 (Table 1), due to the higher values of R^2^ and lower values of χ^2^. On the other hand, even considering 200 terms of the series, the statistical indicators show that model 2 has a slight reduction in the quality of fit to the experimental data at temperatures of 70 and 80 °C, which indicates that water transport was controlled not only by internal resistance, but also by the influence of external resistance to mass transfer. These results suggest that the drying temperature influences the external structure of the sample, modifying the most appropriate boundary condition for the solution of the diffusion equation (Equation (1)). Although the increase in drying temperature accelerates the removal of water, the surface of the product dries faster than the center and forms a less permeable layer, increasing the resistance to heat transfer to the samples and establishing a barrier to mass flow [65,66]. As a consequence, the boundary condition of the first kind becomes physically inadequate to describe drying at high temperatures and model 2 should also be avoided for describing the drying of cumbeba waste. This observation becomes even more evident in the analysis of Figure 2. In this figure, the simulated curves, at the initial instants, are below the experimental points and, after a certain time, are always above these points until the end of the process, which, according to [67], is a typical behavior of a diffusion phenomenon in which a certain resistance occurs on the surface (limit) of the product, but has not been considered in the solution of the governing differential equation, as in the case of model 2. This result is in accordance with observations from previous studies [35,36,51,55,68].

### 3.3. Results Obtained with the Model 1

For the third-kind boundary condition (model 1), the results obtained for all temperatures are presented in Table 3. The statistical indicators obtained can be considered reasonable (R^2^ ≥ 0.9951 and χ^2^ ≤ 1.9738 × 10^−2^) for all temperatures investigated. A comparison between the models, for the drying process at 80 °C, indicates that the chi-square for model 1 is 5.39-times lower than this statistical indicator for model 2. In the comparison of models 1 and 3, this factor is 11.58. These comparative factors serve to indicate that model 1 represents a better fit than those of models 2 and 3. In addition, the coefficients of determination obtained for model 1 are higher than the values obtained by the other models (see Table 3). Model 2 presents coefficients of determination close to the corresponding values of model 1, but always slightly lower. As the domain of this statistical indicator is between 0.0 and 1.0, each significant figure of R^2^ is important, and model 1 really represents the experimental data better. This statement is even more evident when looking at chi-squares: model 2 has chi-squares with values that are about five times the value corresponding to model 1. Thus, among the three models analyzed in the present study, model 1 is the most indicated to describe the convective drying of cumbeba waste. This result is in accordance with observations of previous studies on the drying of different types of biological products, where the model that considers third-kind boundary conditions also describes the experimental data better [34,35,36,37,38].

**Figure 3:**
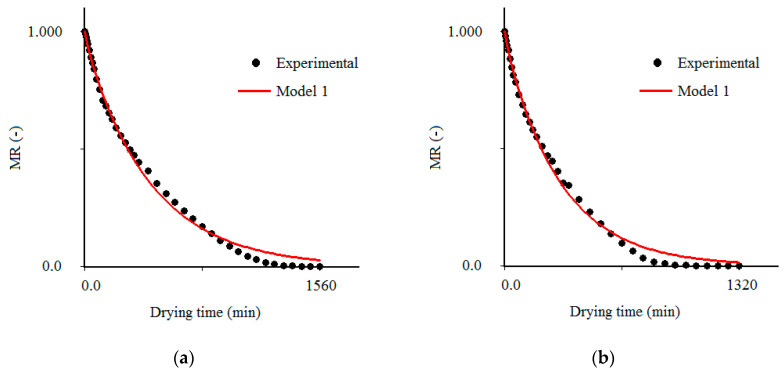

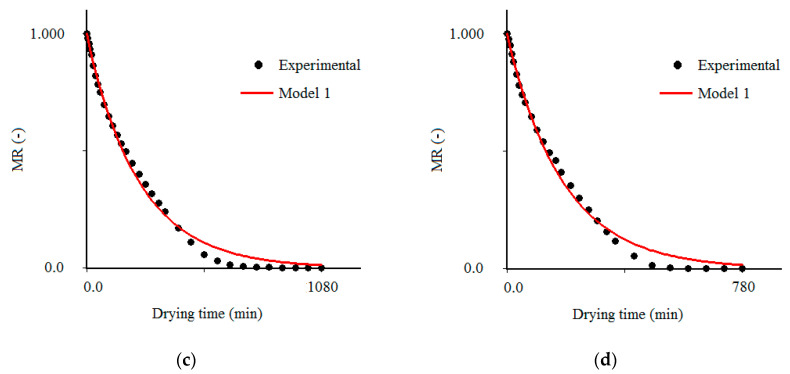
Comparison of dimensionless moisture contents obtained in the experiment and predicted using model 1 at drying air temperatures of (**a**) 50 °C, (**b**) 60 °C, (**c**) 70 °C and (**d**) 80 °C.

As the process parameters (*D_ef_*, *Bi* and *h*) were determined via optimization technique, Equation (9) was used in the simulations of the drying kinetics of the experimental data sets, for the temperatures of 50, 60, 70 and 80 °C, as shown in Figure 3. An analysis of Figure 1, Figure 2 and Figure 3 reveals the same conclusion, that model 1 results in a better fit to experimental data than models 2 and 3. The low values of Biot number and mass transfer obtained through model 1, under different experimental conditions, presented in Table 3, confirm the existence of a resistance to mass flow (water) on the surface of the product, mainly in drying at 70 and 80 °C. However, even model 1, which considers that there is some external resistance to water transport and has reasonable statistical indicators, cannot accurately capture the physical nature of the drying process of cumbeba waste.

In Figure 3a–d it is possible to observe that, at the beginning of the process, the simulated curve fits well to the experimental points, but at the end it continues above these points. An explanation for this result is that the model analyzed (model 1) has restrictive conditions when it does not consider that during drying the properties of heat and mass transport—thermophysical parameters—may vary due to heterogeneities and anisotropic behavior of the materials [69,70], structural changes associated mainly with shrinkage [71,72] and also to the temperature and/or composition of the material [25,48,51,73,74,75]. In addition, the combination of temperature and humidity during drying has an influence on the glass transition of the material. With the drying time, the water content decreases and the glass transition temperature (Tg) rises. With this, the material can change from the elastic state to the glassy state, with the diffusion decreasing until reaching the equilibrium water content [76]. Therefore, for a rigorous description of the drying process, these factors should be considered. In this case, it is clear that the analytical solution of the diffusion equation presented in this article is not a fully appropriate model to describe the actual drying behavior of cumbeba waste. The results obtained in the present study can serve as initial values for processes of optimization of mass transport properties (*D_ef_* and *h*) that involve a more realistic description of the physics of the drying process of cumbeba waste, such as those that use models obtained from numerical solutions of the diffusion equation that consider the influence of deformations by contraction and/or variation of composition (moisture content) on transport phenomena [77,78,79,80].

**Figure 4:**
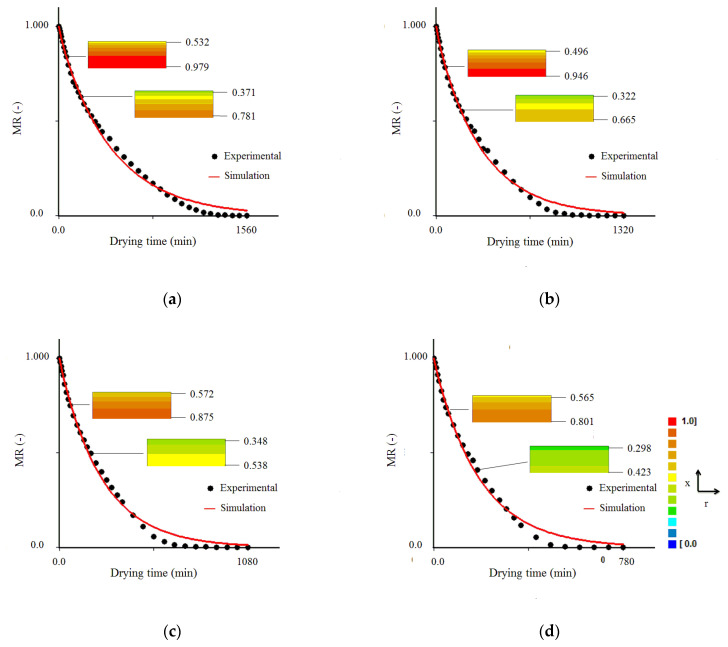
Cumbeba waste drying kinetics at drying temperatures of (**a**) 50 °C, (**b**) 60 °C, (**c**) 70 °C and (**d**) 80 °C, highlighting the distribution of dimensionless moisture content (*MR*) at *t* = 60 min and *t* = 180 min. Rectangles are not at scale. (For interpretation of the references to color in this figure legend, the reader is referred to the web version of this article.).

Once the parameters of drying kinetics are calculated (Table 3), Equation (9) makes it possible to determine the dimensionless moisture content (*MR*) at any point of the sample, at a previously stipulated instant. The experimental data and the fitting curves of the drying kinetics for all tests are presented in Figure 4. This figure also shows the distribution of the dimensionless moisture content at instants *t* = 60 min and *t* = 180 min for all drying temperatures. The rectangles that show the water distribution, as a function of *MR*, in the cross-section of the infinite slab, which represents the cumbeba waste sample, show the maximum and minimum values of the dimensionless moisture content at the base (*x* = 0) and surface of the sample (*x* = *L*), respectively.

In Figure 4, at *t* = 780 min, the mean values of dimensionless moisture content obtained by simulation were 0.16, 0.08, 0.04 and 0.02, for temperatures of 50, 60, 70 and 80 °C, respectively, reflecting the increase imposed by temperature rise on the speed of the water removal process, reducing drying time, as observed in previous studies [26,42,81]. This means that the effective mass diffusivity is lower in drying at low temperatures, as predicted by model 1 (see Table 3), which promotes the formation of a high water gradient in the sample, making the drying process less homogeneous and longer (long drying time). In addition, in Figure 4 it is possible to observe that the local moisture content has continuously decreased with the drying time. As an example, for the temperature of 50 °C, at *t* = 60 min, the maximum and minimum values of the dimensionless moisture content were 0.979 (base) and 0.532 (surface) and, at instant *t* = 180 min, these values were 0.781 (base) and 0.371 (surface) (see Figure 4a).

**Figure 5:**
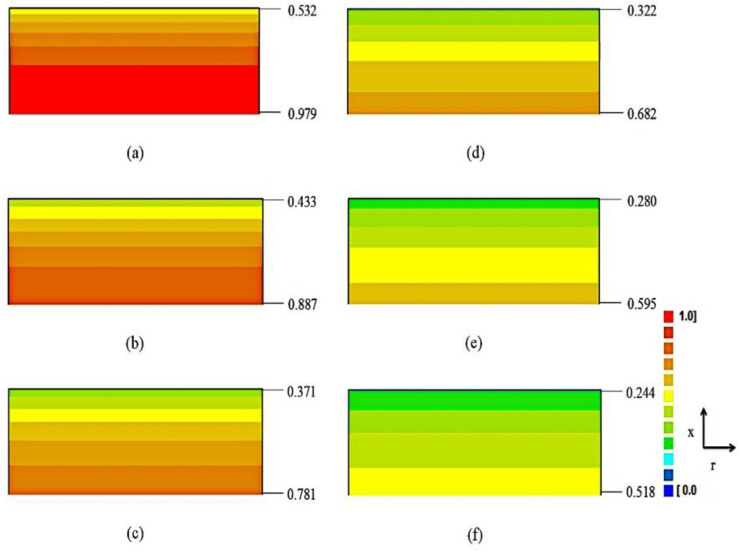
Distributions of dimensionless moisture contents (*MR*) for drying at 50 °C a: (**a**) *t* = 60 min; (**b**) 120 min; (**c**) 180 min; (**d**) 240 min; (**e**) 300 min and (**f**) 360 min. Rectangles are not at scale. (For interpretation of the references to color in this figure legend, the reader is referred to the web version of this article.).

The distributions of dimensionless moisture content for the temperature of 50 °C at six instants are shown in Figure 5, where it is possible to note that the water flow traveled from the aluminum tray-waste interface (*x* = 0) to the sample surface (*x* = *L*) exposed to air. Over time (Figure 5a–f), the external layers of the waste become increasingly unsaturated by moisture, with progressive reduction in moisture content gradient, and it is possible to observe a drying front moving from the surface to the base of the product. Similar results have been found in previous studies, where the presence of residual moisture inside the sample was also observed experimentally and/or by simulation, while its surface was virtually dry [79,82,83,84,85,86]. From Figure 5 it can be concluded that, even with limitations, the diffusion model presented in this study (model 1) is a useful tool to predict the moisture distribution of cumbeba waste at a given time, enabling the study of several scenarios without the need for new experiments.

**Figure 6:**
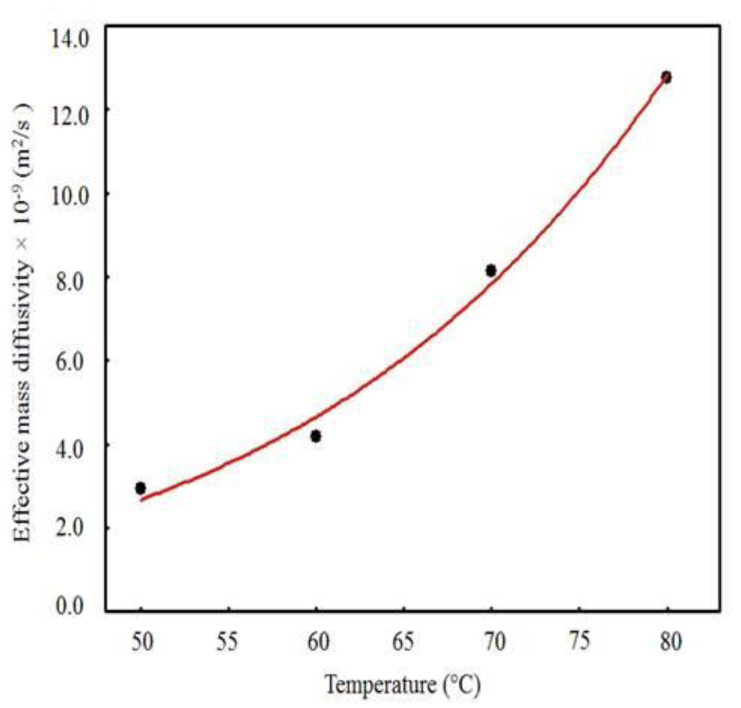
Effective mass diffusivity versus drying air temperature.

**Figure 7:**
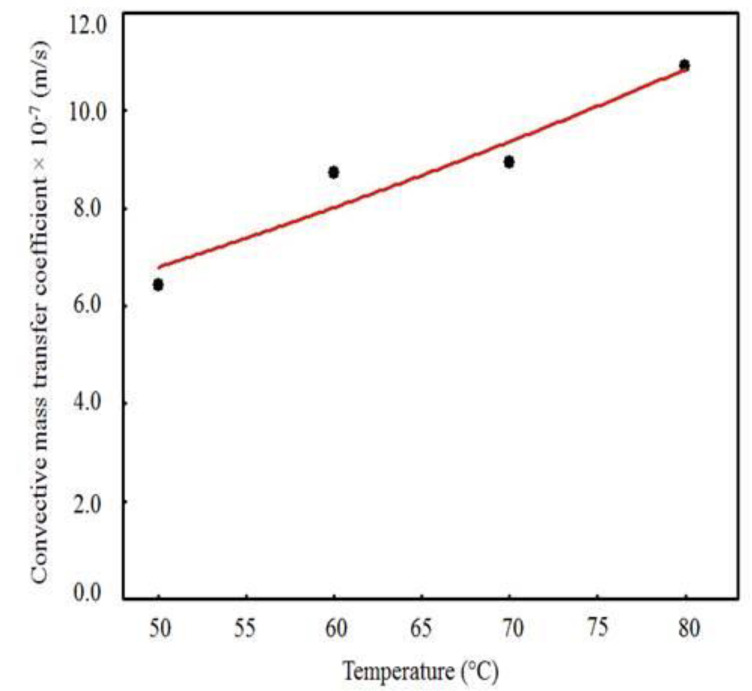
Convective mass transfer coefficient versus drying air temperature.

The values of *D_ef_* and *h* obtained from the simulations (Table 3) as a function of the drying temperature, as well as the curves for these parameters obtained from the Arrhenius-type equations (Equations (17) and (18)), are shown in Figure 6 and Figure 7. There is a tendency of increase of *D_ef_* and *h* with the increase in temperature (Figure 6 and Figure 7, respectively), which is explained by the increase in the rate of heat transfer between the waste and the drying air, caused by the increase in temperature, which results in greater agitation of water molecules [57,58], facilitating water diffusion to the sample surface and removal by the drying air. The *D_ef_* values obtained in this study (2.9285 × 10^−9^–1.2754 × 10^−8^ m^2^/s) are similar to the values of diffusivity reported for fruits and legumes, from 10^−11^–10^−9^ m^2^/s [87].

With the fit of Equation (17) to the pairs (*T*, *D_ef_*), presented in Table 3, the following results were obtained: *D*_0_ = 1.0238 × 10^−1^ m^2^/s and *E_a_*/*R* = 5618.416 K, with a coefficient of determination of 0.993. Thus, Equation (17) can be rewritten as:

(19) $$D_{ef}\;=\;1{.0238\times 10}^{-1}\;exp\left[-\frac{5618.416}{\left(T\;+\;273.15\right)}\right]$$

The fit of Equation (18) to the pairs (*T*, *h*) presented in Table 3 leads to the following results: *A* = 1.671 × 10^−4^ m^2^/s and *B* = *E_a_*/*R* = 1778.955 K, with a coefficient of determination of 0.919, which was considered adequate, given the great natural variability of the raw material. Thus, Equation (18) can be rewritten as:

(20) $$h\;=\;1.671\times 10^{-4}\;exp\left[-\;\frac{1778.955}{\left(T\;+\;273.15\right)}\right]$$

The activation energies of water diffusion and convection of cumbeba waste were calculated using Equations (19) and (20), respectively. The activation energy (*E_a_*) values under the different drying conditions were 46.71 kJ/mol and 14.790 kJ/mol for diffusion and convection, respectively. These values are within the range of activation energy reported for different biological materials, between 12.7 and 110 kJ/mol [88].

## 4. Conclusions

The comparison between the simulations and the experimental data made it possible to conclude that model 1, which considers the internal diffusive mass flow equal to the external convective flow in the vicinity of the samples (boundary condition of the third kind) for the solution of the diffusion equation, is the most appropriate to describe the drying process of cumbeba waste. At the lowest drying temperatures (50–60 °C), an equilibrium condition on the sample surface (boundary condition of the first kind), for the solution of the diffusion equation (model 2) satisfactorily describes the drying kinetics of the cumbeba waste (R^2^ between 0.995–0.996), however even in these cases, model 1 presents the best statistical indicators (R^2^ > 0.996). The higher the temperature, the higher the diffusivity of water and the shorter the time required for the waste to reach the equilibrium moisture content, as predicted by model 1. Although model 1 is useful to estimate the temporal and local evolution of water distribution during the drying of cumbeba waste, for a better understanding of the process, in future experiments, heterogeneities and anisotropies of transport properties (*D_ef_* and *h*), as well as contraction effects, need to be considered. For this purpose, simulation with numerical models can provide a more complete view of the transport phenomena of the drying process of cumbeba waste. Under the studied conditions, it was possible to obtain empirical equations for the process parameters (*D_ef_* and *h*) as a function of the drying air temperature, which makes it possible to predict the kinetic behavior at a temperature chosen within the range from 50 to 80 °C, without the need to perform new experimental tests.

## Figures and Tables

**Table 1:** Effective mass diffusivity for model 3.

Temperature (°C)	Effective Mass Diffusivity (*D_ef_*) (m^2^/s)	R^2^	χ^2^ × 10^−1^
50	1.1303 × 10^−9^	0.9380	3.1172
60	1.5615 × 10^−9^	0.9442	2.5671
70	1.9665 × 10^−10^	0.9386	2.5427
80	2.5440 × 10^−10^	0.9324	2.2856

**Table 2:** Effective mass diffusivity for model 2.

Temperature (°C)	Effective Mass Diffusivity (*D_ef_*) (m^2^/s)	R^2^	χ^2^ × 10^−1^
50	1.1285 × 10^−9^	0.9959	1.2094
60	1.5603 × 10^−9^	0.9954	1.0265
70	1.9615 × 10^−9^	0.9934	1.1395
80	2.5368 × 10^−9^	0.9921	1.0645

**Table 3:** Effective mass diffusivity, convective mass transfer coefficient and Biot number for model 1.

Temperature (°C)	Effective Mass Diffusivity (*D_ef_*) (m^2^/s)	Convective Mass Transfer Coefficients (*h*) (m/s)	Biot Number (Bi) (-)	R^2^ (-)	χ^2^ × 10^−2^ (-)
50	2.9285 × 10^−9^	6.4362 × 10^−7^	2.00	0.9962	2.2826
60	4.1695 × 10^−9^	8.7273 × 10^−7^	2.00	0.9962	2.1171
70	8.1395 × 10^−9^	8.9445 × 10^−7^	1.05	0.9957	2.1434
80	1.2754 × 10^−8^	1.0912 × 10^−6^	0.82	0.9951	1.9738
